# Density Underpins Shifts From Competition to Facilitation in Co‐Invading Blowflies

**DOI:** 10.1002/ece3.74251

**Published:** 2026-09-10

**Authors:** Daniel Nunn, Nathan J. Butterworth, Angela McGaughran

**Affiliations:** ^1^ Te Aka Mātuatua ‐ School of Science University of Waikato Hamilton New Zealand; ^2^ School of Life and Environmental Sciences Deakin University Burwood Victoria Australia

**Keywords:** developmental limitation, Diptera, facilitation, interspecific interactions, larval dynamics

## Abstract

The success of an invasive species is driven by its interactions with both native species and co‐occurring invaders. These interactions can range from competitive exclusion to mutual facilitation. However, the factors that govern the direction of species interaction outcomes remain poorly understood in invasive systems, including their environmental dependency. We performed species interactions experiments between two co‐occurring invasive blow flies: *Calliphora stygia* and *Lucilia sericata*. We varied both total larval density (*n* = 10, 20, 30, 50) and relative species ratios (100%, 70%, 50%, 30%) and measured outcomes in the form of adult emergence success and adult body size. We found that interaction outcomes varied by species. For intraspecific experiments, *C. stygia* experienced more pronounced effects of competition, with a more than 2.4‐fold greater reduction in body size between low (*n* = 10) and high (*n* = 50) densities than experienced by *L. sericata* between the same densities. *Calliphora stygia* also experienced reduced adult emergence at *n* = 30 and *n* = 50, while *L. sericata* emergence was not affected. However, interspecific competition drove contrasting density‐dependent species‐specific responses. At low density interspecific ratios of 70% and 50%, female *C. stygia* performance reflected facilitation (i.e., increased body size relative to intraspecific competition), with no effect for *L. sericata*, while increasing density shifted interaction dynamics towards competition (i.e., interspecific and intraspecific effects became comparable) for both body size and adult emergence under all species ratios except 50C:50 L for males, where *C. stygia* body size decreased. In contrast, *L. sericata* performed worse under all high density interspecific conditions relative to intraspecific competition when measuring body size, suggesting stronger interspecific effects, regardless of species ratios. The asymmetric response of these two co‐occurring blow flies highlights the nuance of co‐invader interactions: competition outcomes between invaders are not fixed but rather strongly shaped by both density and species balance.

## Graphical abstract

We performed species interactions experiments on > 1900 individuals of co‐occurring invasive blowflies. We found that interaction outcomes varied by species, suggesting that competitive outcomes for co‐invaders are strongly shaped by both density and species balance.

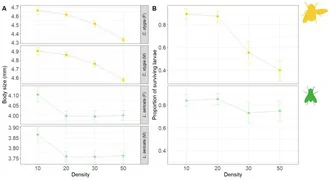

## 1 Introduction

Biological invasions represent one of the most pressing threats to global biodiversity, with over 37,000 invasive species documented worldwide (IPBES 2024). Through reductions in native species populations, disruption of ecosystem functioning and negative impacts on human well‐being, their consequences are far‐reaching and potentially irreversible (Ehrenfeld 2010; Pyšek et al. 2020). Predicting and managing invasion success requires mechanistic understanding of what determines whether an invader establishes, spreads, or fails (Lennox et al. 2015; Thompson et al. 2021).

Species interactions are central to invasion outcomes, as reflected by two prominent hypotheses—the *biotic resistance hypothesis*, which posits that native species diversity constrains the niche space available to invaders (Beaury et al. 2020); and the *invasional meltdown hypothesis*, which suggests that invasive species can facilitate one another through a self‐reinforcing cycle of establishment, spread and impact (Ricciardi and Simberloff 2025; Simberloff and Von Holle 1999). Although research often focuses on invasive species outcompeting natives (Carmo et al. 2018; Cavraro et al. 2022; Colléony and Shwartz 2020; Cortes et al. 2016; Gabel et al. 2024; Lele and Pârvulescu 2017), interactions between invasive species are also important drivers of invasion success (Jackson 2015). Indeed, co‐invaders can facilitate one another's spread. For example, Argentine ants ( *Linepithema humile* ) and *Acacia* spp. co‐invaded South Africa through mutualistic seed dispersal, accelerating their joint range expansion (Devenish et al. 2025). Co‐invaders can also suppress one another through competitive exclusion. An example is seen in southern Chile, where populations of *Oncorhynchus mykiss* (rainbow trout) and *Salmo trutta* (brown trout) have constrained the establishment of subsequently arriving invasive salmonids (Arismendi et al. 2014).

Although the biotic resistance and invasional meltdown hypotheses both position interaction outcomes as fixed, such outcomes may be expected to be variable in cases where co‐invaders share novel environments without the evolutionary history of co‐occurrence that structures native communities (Fernández and Hamilton 2015; Mooney and Cleland 2001), and where they frequently occupy overlapping ecological niches (Balzani et al. 2020; Jackson and Britton 2014; Quinn et al. 2014). However, the factors that govern whether species interactions are facilitative or competitive, as well as how fixed versus flexible (i.e., environmentally contingent) they are, remain poorly understood (Móréh et al. 2024), limiting our ability to predict invasion outcomes.

Density dependence is likely to strongly shape co‐invader interactions. The *stress‐gradient hypothesis* predicts a shift from competition to facilitation as environmental stress, such as crowding, increases (Bertness and Callaway 1994; Rius and Mcquaid 2009) and empirical work across a range of systems supports a density‐mediated transition between these interaction types (Alto et al. 2015; Hart 2023; Tsurim et al. 2013). This dynamic is especially relevant for invasive populations, where density fluctuates dramatically across invasion stages (Barney et al. 2016; Travis et al. 2009), meaning the directionality of interaction outcomes between the same pair of co‐invaders may shift from competitive to facilitative as an invasion progresses. However, the role of density dependence in co‐invader interaction outcomes remains largely unaddressed despite its likely relevance to how invasion dynamics unfold and change across time and space.

Carrion‐breeding blow flies (Calliphoridae) offer an exceptional model system for addressing questions about invasive species interactions and their density dependence. Carrion is a nutrient‐rich but ephemeral resource that concentrates ecological dynamics in time and space, generating intense intra‐ and interspecific competition among carrion‐breeding species (Prinkkilá and Hanski 1995; Shorrocks et al. 1979). Blow fly larvae engage in collective exodigestion—releasing enzymes that liquefy carrion and enhance resource accessibility for both conspecifics and heterospecifics (Evans 1956; Scanvion et al. 2018) and larger aggregations generate elevated microclimatic temperatures that increase larval development rates (Charabidze et al. 2011; Scanvion et al. 2018). These facilitative mechanisms are intrinsically density‐dependent: at low densities, larvae gain insufficient thermal and enzymatic benefit, with downstream reductions in survival and adult body size (Goodbrod and Goff 1990). Blow flies therefore offer a system in which the mechanistic basis for a density‐mediated shift from facilitation to competition could be elucidated.

**FIGURE 1:**
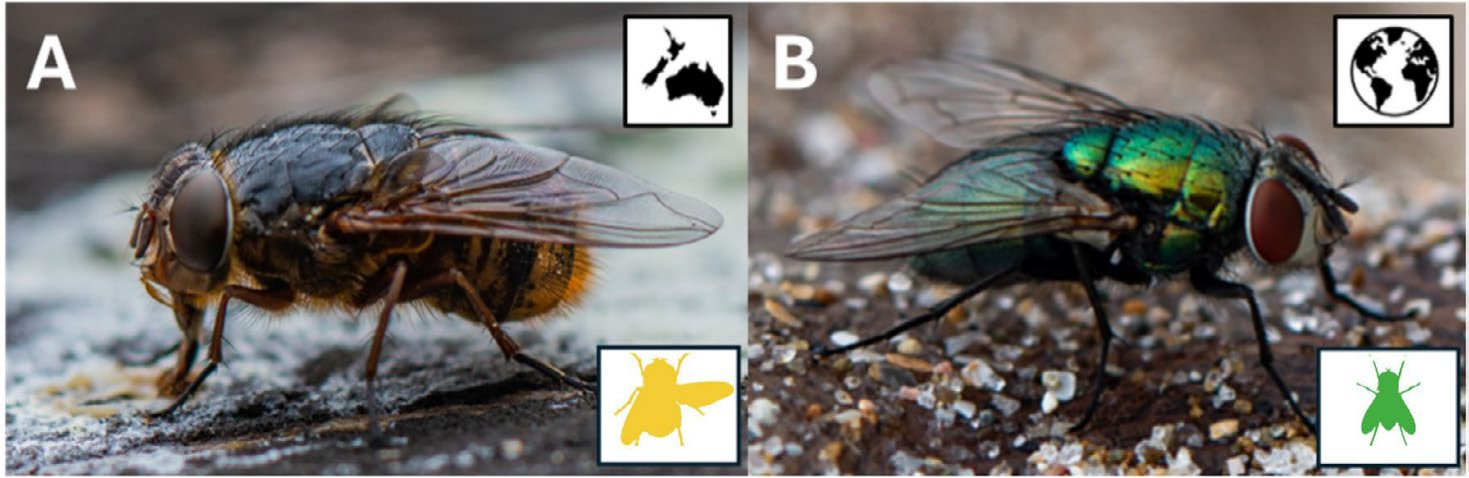
The two focal species of the study. (A) *Calliphora stygia* (Diptera: Calliphoridae), photographed feeding on bird dung at Wairēinga, Waikato, New Zealand; and (B) *Lucilia sericata* (Diptera: Calliphoridae), photographed on a of tetraodontid carcass at Whangamatā beach, Waikato, New Zealand. Photos credited to Nathan Butterworth. The inset images show the geographic distribution (Australasia or cosmopolitan) and silhouettes of each species (the yellow fly icon represents *C. stygia* , while the green icon represents *L. sericata* and these are approximately scaled by adult body size).

Multiple invasive blow fly species co‐occur in New Zealand, providing a tractable system for investigating co‐invasion dynamics. *Calliphora stygia*, an obligate carrion breeder native to Australia, arrived in New Zealand between 1779 and 1841, while the cosmopolitan *Lucilia sericata* established approximately a century later, around 1979 (Dear 1986) (Figure 1). Using controlled laboratory larval rearing experiments, we manipulated total larval density and intra‐ versus interspecific ratios to quantify their independent and interactive effects on larval survival and adult body mass. Consistent with the stress‐gradient hypothesis and the known facilitative properties of larval aggregations, we predicted that both species would show a pattern in which facilitation predominated at high densities (where resource depletion and crowding occur), with this shifting to competition at low densities (where larvae gain insufficient collective benefit from each other's presence). By testing this non‐linear density‐dependence, we aimed to provide direct insight into how the balance between facilitation and competition among co‐invaders shifts contextually, with broad implications for predicting the trajectory of co‐invasions in the wild.

## 2 Materials and Methods

Blow flies were collected from the Waikato region of New Zealand from January to May 2024. *Lucilia sericata* was collected at the University of Waikato campus (GPS coordinates: −37.789354, 175.316167), while *C. stygia* was collected at the University of Waikato campus and at two additional sites: Newstead Gully (−37.7759089, 175.358490) and the Edmund Hillary Hope Reserve (−37.844277, 175.070460). Although *C. stygia* was collected from several sites, previous genetic analysis has shown an absence of genetic structuring among populations from across the North Island (Croft et al. 2024), suggesting sampling represents a single panmictic population.

Prepared baits were used at sampling sites to attract target species, with blow flies then caught on the meat and surrounding vegetation using hand nets. Species were preliminarily identified following Dear (1986) and transferred into a mesh cuboid insect cage (W32.5 x D32.5 x H32.5 cm), which was transported by car (up to 45 min) to the invertebrate laboratory at the University of Waikato. Laboratory conditions consisted of a constant temperature (22°C ± 1°C) and 12:12 h day: night light cycling. Raw sugar and water were provided *ad libitum* to cages, and all flies underwent an initial 24‐h acclimation period in cages shared with other blow flies that had been captured on the same day.

Following the acclimation period, females of each species were isolated into new mesh cages (L21 × W21 × H6 cm) with *ad libitum* sugar and water, and 50 g of beef mince added to encourage egg‐laying. This decision was predicated on the assumption that females had already mated either prior to collection or during the acclimation period when they were still housed alongside males. *Lucilia sericata* was isolated as single females, but *C. stygia* required other females (*n* = 5) to be present to encourage consistent egg‐laying. *Calliphora stygia* adult females were identified (following Dear 1986) prior to their isolation in groups. Isolated individual *L. sericata* females were identified after detection of oviposition. After approximately 48 h, all cages were checked for the presence of larvae, at which point the female adult was removed. Second instar larvae were then age‐matched and selected for rearing trials. A total of 1960 larvae were selected and used in our trials.

To examine how species composition and larval crowding affect blow fly fitness, we set up assays using four larval densities (*n* = 10, 20, 30 and 50 larvae per container) and a fixed amount of beef mince (20 ± 0.25 g). Thus, as larval numbers increased, less food was available per larva (approximately, 2.00, 1.00, 0.67 and 0.40 g per larva, respectively). Five species ratio treatments were tested: 100% *C. stygia* (FC), 100% *L. sericata* (FL), an equal 50:50 mix (50C:50 L), a 70:30 mix favouring *L. sericata* (70% L) and a 70:30 mix favouring *C. stygia* (70% C). Size‐matched second instar larvae were transferred into the meat at the assigned densities and ratios using a fine‐tipped paintbrush. Each container of meat and larvae was then placed inside a polypropylene box (21 × 21 × 6 cm) filled with chaff to provide a pupation substrate. Total replication numbers can be found in Table 1.

**TABLE 1:** Total number of replicate experiments performed for various total larval densities and species ratios. FC = 100% *C. stygia* , FL = 100% *L. sericata* , 70% L = 70% *L. sericata* and 30% *C. stygia* , 70% C = 70% *C. stygia* and 30% *L. sericata* , 50C:50 L = 50% *C. stygia* and 50% *L. sericata* .

Total larval densities	Density ratios					
	FC	FL	50C:50 L	70% L	70% C	Total flies
10	5	7	5	3	5	250
20	5	7	NA	NA	NA	140
30	5	4	NA	NA	NA	370
50	5	4	5	4	6	1200

All boxes were checked daily for blow fly emergence. Emergence success was measured by calculating the number of larvae from the initial species density for which adult blow flies emerged following pupation. Finally, within 48 h of emergence, blow flies were euthanised by freezing, after which adult body size was measured as a proxy for fitness. Previous work has shown that even small changes in body size correlate with significant reductions in sperm/eggs for blow flies; (Saunders and Bee 2013; Wall 1993). To measure body size, dead blow flies were positioned horizontally and pierced by a small metal pin, allowing a consistent view of the head and thorax. Pinned flies were then placed under a stereo microscope equipped with a Canon EOS 600D camera and photographed from above. ImageJ v.0.5.8 (Schneider et al. 2012) was then used to measure the length and width of the thorax and length of the head to obtain a representative body size measurement per individual (e.g., Croft et al. 2024).

All data were analysed in R v.4.3.3 (R Core Team 2024) and plotted using ggplot2 v.3.5.1 (Ginestet 2011). A Pearson's moment correlation test was first used to assess the relationship between thorax length and overall body size (assessed by multiplying head length, thorax width and thorax length). A correlation was confirmed for both species: *L. sericata* = 0.883, *p* < 0.001; *C. stygia* = 0.904, *p* < 0.001, hence subsequent analyses used only the thorax length measurement as a proxy for body size. The effects of species ratio and density on thorax length were assessed using linear models fit with density, species ratio and sex as predictors. Effect sizes were generated as a product of estimates plus standard error to form 95% confidence intervals; all derived from linear models. Differences in survival rates among treatments were tested as the proportion of emerging adults per assay and fit to a beta regression using the betareg v.3.2–0 package (Cribari‐Neto and Zeileis 2010) and secondly, as successes and failures in emergence using a glm fit to a binomial distribution. Instances of over‐dispersion were detected and overcome for binomial models by fitting to a quasi‐binomial distribution. Estimated marginal means analysis was conducted using the emmeans v.1.10.0 (Lenth et al. 2019) to test pairwise comparisons of thorax length and survival rates between density and species ratio levels.

## 3 Results

### 3.1 Species‐Specific Responses to Intraspecific Density

**FIGURE 2:**
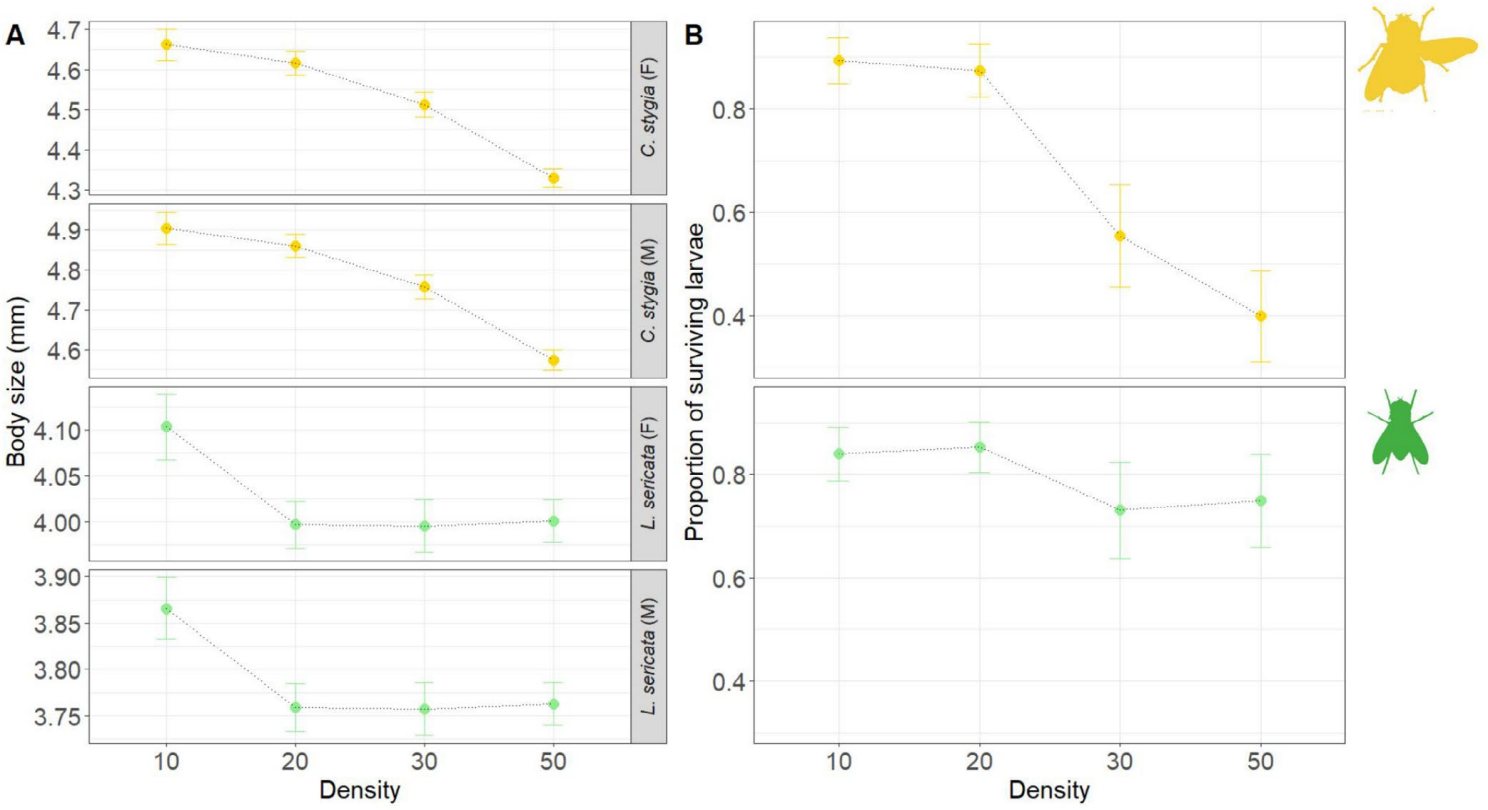
The effects of density under full intraspecific conditions for *Calliphora stygia* and *Lucilia sericata* on: (A) male and female thorax length; and (B) adult emergence (measured as proportion of surviving larvae). The yellow fly icon represents *C. stygia* , while the green icon represents *L. sericata* and these are approximately scaled by adult body size. Sex was only assigned to thorax length measurements because determination of larval sex was not possible. Error bars indicate 95% confidence intervals.

We observed species‐specific responses to increasing intraspecific densities, with *C. stygia* experiencing more severe reductions in body length and *L. sericata* showing only marginal changes. Specifically, *C. stygia* showed thorax length reductions ranging between 0.046 mm (*n* = 10 to *n* = 20) and 0.103 mm (*n* = 20 to *n* = 30) as density increased (Figure 2A, Table 2). This equated to a reduction of 6.93% and 7.08% in thorax length for males and females, respectively, when comparing densities of *n* = 10 and *n* = 50. For *L. sericata* , significant thorax length reductions were only observed at low density (*n* = 10) compared to all other densities (Figure 2A, Table 2). However, these were on a much smaller scale than those seen in *C. stygia* —with only a 2.84% and 2.44% reduction in thorax length for males and females, respectively. No reductions in body size past a density of *n* = 20 were observed for *L. sericata*, meaning *C. stygia* experienced at minimum 2.4× stronger reductions in thorax length over all assays.

**TABLE 2:** Pairwise comparisons of thorax length under varying intraspecific density conditions from estimated marginal means modelling for *Calliphora stygia* and *Lucilia sericata*. Thorax length comparisons were derived from linear regression models testing the effects of density, sex and species. Significant effects lower than *p* < 0.05 are indicated in bold.

Density comparison	Estimate	Standard error	T value/ratio	*p*
***Calliphora stygia***				
*n* = 10 vs. *n* = 20	0.046	0.039	0.990	0.756
*n* = 10 vs. *n* = 30	0.443	0.062	7.147	**< 0.001**
*n* = 10 vs. *n* = 50	0.606	0.067	9.072	**< 0.001**
*n* = 20 vs. *n* = 30	0.103	0.038	2.749	**0.031**
*n* = 20 vs. *n* = 50	0.286	0.033	8.570	**< 0.001**
*n* = 30 vs. *n* = 50	0.183	0.034	5.383	**< 0.001**
***Lucilia sericata***				
*n* = 10 vs. *n* = 20	0.106	0.039	2.705	**0.035**
*n* = 10 vs. *n* = 30	0.109	0.041	2.619	**0.044**
*n* = 10 vs. *n* = 50	0.103	0.038	2.715	**0.034**
*n* = 20 vs. *n* = 30	0.002	0.034	0.061	1.000
*n* = 20 vs. *n* = 50	−0.004	0.030	−0.120	0.999
*n* = 30 vs. *n* = 50	−0.006	0.033	−0.174	0.998

Adult emergence at the various intraspecific densities showed species‐specific patterns consistent with the thorax length results. For *C. stygia* , adult emergence was reduced at densities of *n* = 30 and *n* = 50 when compared to a density of *n* = 10. In contrast, *L. sericata* displayed no significant changes in adult emergence with density under full intraspecific conditions (Figure 2B).

### 3.2 Density Dependence Underpins Shifts From Facilitation to Competition Between Co‐Invaders

**FIGURE 3:**
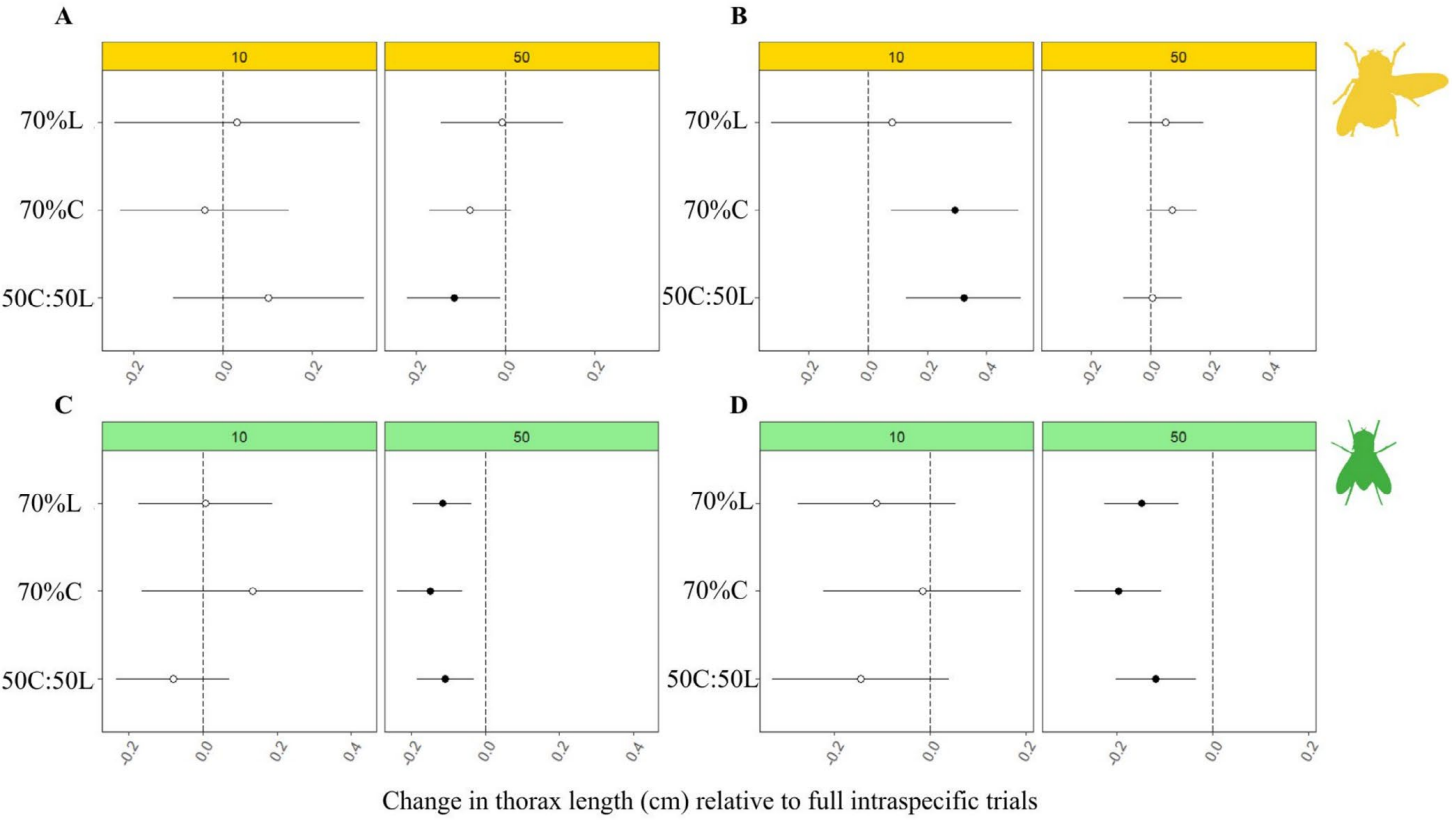
Effects of species ratios on thorax length for *Calliphora stygia* and *Lucilia sericata*: (A) males; and (B) females. All panels present comparisons with low (*n* = 10) and high (*n* = 50) density treatments, with effect sizes calculated relative to thorax length under full intraspecific density (as represented by the dotted base line). The yellow fly icon represents *C. stygia* , while the green icon represents *L. sericata* and these are approximately scaled by adult body size. Significant effects are indicated when 95% confidence intervals do not overlap zero and are displayed in bold. Confidence intervals positioned to the right of zero indicate positive effects on interspecific ratios on thorax length, whereas those to the left indicate negative effects.

At low larval densities (*n* = 10), interspecific competition had a significant positive effect on *C. stygia* thorax length, indicative of a facilitatory interaction. Compared to the full intraspecific *C. stygia* treatment, both the equal mix (50C:50 L) and the *C. stygia* ‐favoured mix (70% C) produced 7.15% and 6.51% larger thorax lengths in female *C. stygia* (Figure 3; Table 3). For *L. sericata* , thorax length was unaffected by species ratio at low density in both sexes (Figure 3; Table 3).

**TABLE 3:** The effect of density and species ratios on thorax length from estimated marginal means modelling based on individual linear models separated by species and sex for *Calliphora stygia* and *Lucilia sericata*. Significant effects lower than *p* < 0.05 are indicated in bold.

Species ratio/density	Estimate	Standard error	*T* value	*p*
***Calliphora** **stygia*** **males**				
50C:50 L/*n* = 10	0.103	0.109	0.944	0.346
70S/*n* = 10	−0.041	0.096	−0.425	0.671
70 L/*n* = 10	0.032	0.141	0.230	0.818
50C:50 L/*n* = 50	−0.116	0.054	−2.158	**0.032**
70S/*n* = 50	−0.079	0.047	−1.674	0.095
70 L/*n* = 50	−0.008	0.070	−0.115	0.909
***Calliphora** **stygia*** **females**				
50C:50 L/*n* = 10	0.322	0.099	3.260	**0.001**
70S/*n* = 10	0.293	0.109	2.672	**0.008**
70 L/*n* = 10	0.080	0.207	0.386	0.700
50C:50 L/*n* = 50	0.006	0.050	0.124	0.901
70S/*n* = 50	0.071	0.044	1.616	0.107
70 L/*n* = 50	0.051	0.064	0.791	0.429
***Lucilia** **sericata*** **males**				
50C:50 L/*n* = 10	−0.081	0.078	−1.040	0.299
70S/*n* = 10	0.134	0.153	0.877	0.381
70 L/*n* = 10	0.007	0.093	0.877	0.381
50C:50 L/*n* = 50	−0.108	0.039	0.071	**0.006**
70S/*n* = 50	−0.151	0.045	−3.314	**0.001**
70 L/*n* = 50	−0.117	0.041	−2.882	**0.004**
***Lucilia** **sericata*** **females**				
50C:50 L/*n* = 10	−0.146	0.095	−1.544	0.124
70S/*n* = 10	−0.016	0.106	−0.154	0.878
70 L/*n* = 10	−0.112	0.084	−1.327	0.185
50C:50 L/*n* = 50	−0.119	0.043	−2.769	**0.006**
70S/*n* = 50	−0.198	0.046	−4.292	**0.000**
70 L/*n* = 50	−0.149	0.040	−3.720	**0.000**

**FIGURE 4:**
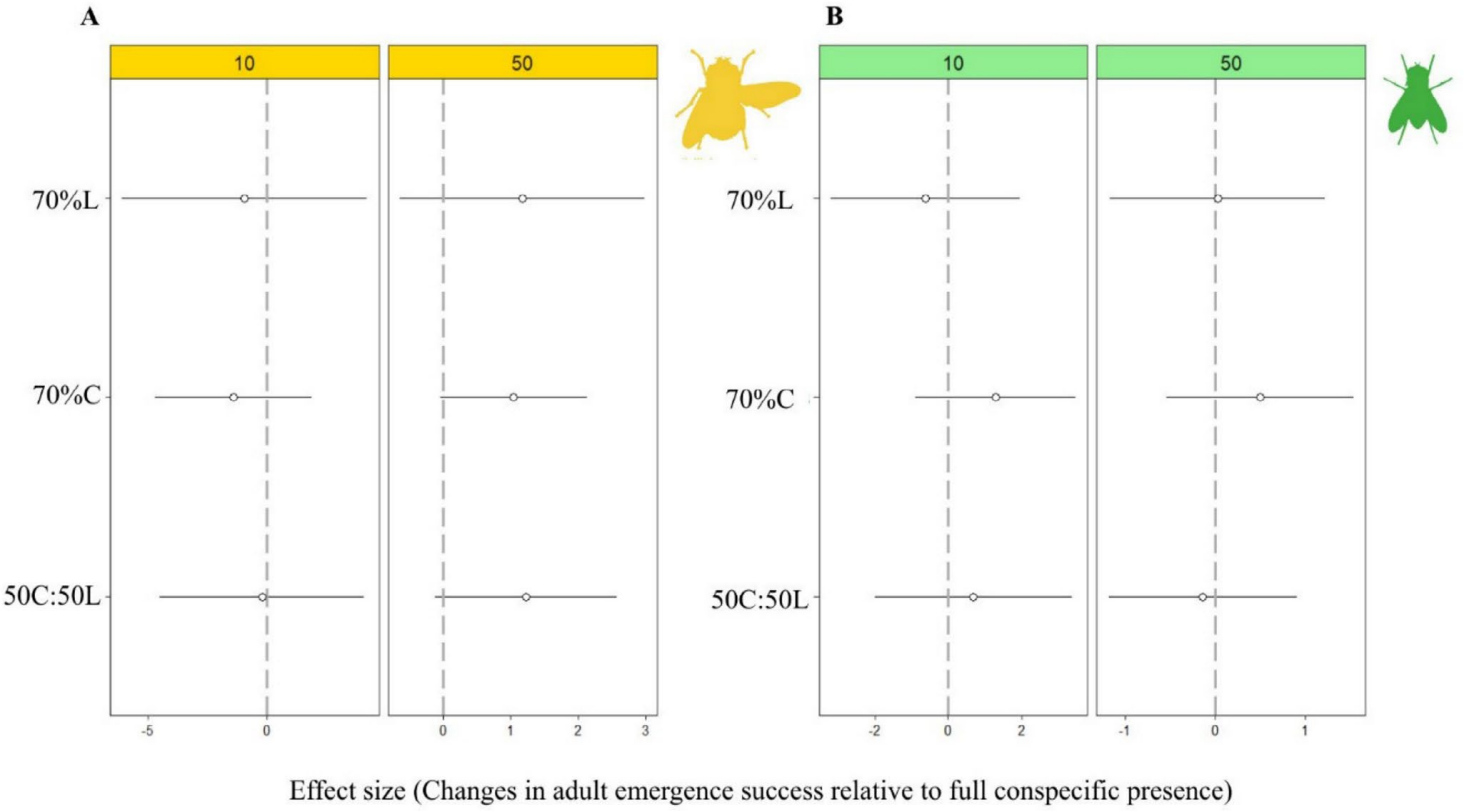
Effects of species ratios on adult emergence in: (A) *Calliphora stygia*; and (B) *Lucilia sericata*. All panels present comparisons within low (*n* = 10) and high (*n* = 50) density treatments, with effect sizes calculated relative to adult emergence success under full intraspecific presence (as represented by the dotted baseline). The yellow fly icon represents *C. stygia* , while the green icon represents *L. sericata* and these are approximately scaled by adult body size. Significant effects are indicated when 95% confidence intervals do not overlap zero. Confidence intervals positioned to the right of zero indicate positive effects on interspecific ratios on adult emergence success, whereas those to the left indicate negative effects.

At high larval densities, competitive effects became more pronounced – particularly for *L. sericata* , which showed significant thorax length reductions between 2.93%–4% for males and 2.99%–5% for females across all three interspecific treatments relative to the intraspecific baseline (Figure 3; Table 3). In contrast, *C. stygia* was less affected. Only males in the 50C:50 L species ratio treatment showed a significant (2.46%) reduction in thorax length, while females were unaffected across all species ratios. Notably, the 50C:50 L species ratio treatment was the only condition in which both species experienced simultaneous reductions in thorax length across sexes, indicating that competitive effects were mutual at equal species balance under high density. Neither larval density nor species ratio had a significant effect on adult emergence success relative to intraspecific trials in either species (Figure 4; Table 3).

## 4 Discussion

We explored how density and species ratios impacted fitness outcomes for co‐invading blow flies and found that density is a key factor mediating the sign and magnitude of interactions between the species, *C. stygia* and *L. sericata* . Our results indicate that facilitation dominates at low density, with a shift towards competition determining interaction outcomes at higher density. Yet this shift was asymmetric across the two species: *C. stygia* was most sensitive to intraspecific competition, while *L. sericata* suffered disproportionately under interspecific competition. Together, these findings demonstrate that density can fundamentally underpin whether co‐invader interactions are competitive or facilitatory.

At low interspecific densities, we found that *C. stygia* females exhibited thorax lengths 6.51%–7.15% larger than intraspecific controls, while *L. sericata* was unaffected—a pattern consistent with interspecific facilitation. One potential explanation for this is that larval aggregations elevate local substrate temperatures through collective thermogenesis, potentially enhancing development with species‐specific effects (Anderson 2000; Bambaradeniya et al. 2019; Charabidze et al. 2011; Charabidze and Aubernon 2023; Komo et al. 2019), while collective exodigestion may increase nutrient availability below densities at which competitive exclusion occurs (Greenberg 1991). The fact that *C. stygia* performed better (i.e., achieved larger increases in body sizes) rather than *L. sericata* —despite the latter having previously been shown to gain body size and survival in mixed‐species cultures (Komo et al. 2019)—suggests that facilitation varies with density and species ratios rather than being a fixed interaction outcome. Why facilitation was restricted to *C. stygia* females specifically remains unclear, but may reflect species differences in feeding behaviour, thermal sensitivity, or genetic traits. Indeed, sex‐specific larval development strategies are not uncommon and have been found among many invertebrate genera, such as *Libellula depressa* (dragonfly; Johannes Mikolajewski et al. 2007), *Melitaea cinxia* (butterfly; Rosa and Saastamoinen 2017), *Aedes triseriatus* (mosquito; Wormington and Juliano 2014) and *Drosophila melanogaster* (fruit fly; Duxbury and Chapman 2020).

Beyond a density threshold of *n* = 10 larvae, we found that competition displaced facilitation, with consequences expressed through reductions in adult body size rather than larval survival. At high density, *L. sericata* experienced thorax length reductions of 2.93%–5% across all interspecific treatments, while *C. stygia* remained largely unaffected. Although < 10% reductions in thorax length may appear modest, their fitness consequences may be substantial. For example, each 0.1 mm reduction in *L. sericata* thorax length equates to an approximate loss of 36 oocytes (Wall 1993), while a 1.0 mm thorax length reduction in *Calliphora* species has been shown to correspond to a 225% decline in egg follicle number (Saunders and Bee 2013). The competitive body size effects documented here are therefore likely to carry meaningful population‐level reproductive consequences.

Analogous density‐dependent shifts from facilitation to competition have been documented in other co‐invader systems, which may suggest that context‐dependence in interaction outcomes is more common than previously thought. For example, *Perna perna* (brown mussel) and *Mytilus galloprovincialis* (Mediterranean mussel) show varying competitive and faciliatory dynamics with varying tidal zone stress (Rius and Mcquaid 2009), while *Z. indianus* (African fig fruit fly) and *D. suzukii* (spotted wing *Drosophila*) switch between facilitation and competition dependent upon fruit substrate and availability (Mccabe and Teets 2025). Notably, competitive and facilitative outcomes were also sex‐specific in our study—facilitation was detected only in *C. stygia* females, and high‐density competitive effects were restricted to males in *C. stygia* . This is likely underpinned by sex‐specific responses to food limitation and maintenance of life history traits (Bedhomme et al. 2003; Duxbury and Chapman 2020; Johannes Mikolajewski et al. 2007; Rosa and Saastamoinen 2017; Wormington and Juliano 2014) and suggests that sex may play an important role in the manifestation of density‐dependent co‐invader interactions. Additionally, we found that *C. stygia* individuals were on average > 20% larger than *L. sericata* (depending on the density and treatment) and were also consistently larger under laboratory conditions in the absence of competition and in wild‐caught individuals (Daniel Nunn, pers. obs.). Thus, relative body size differences between the two species may influence their responses to food limitation, with *C. stygia* likely having higher food requirements during larval feeding stages.

We observed that interspecific interactions were increasingly important under high density for *L. sericata* but had no appreciable effects on *C. stygia* relative to intraspecific competition. This suggests that coexistence may be promoted between the two species under low density and potentially under non‐food limited conditions (though we did not test the latter here since food limitation increased as density increased in our experiments). Indeed, co‐existence should be promoted when intraspecific competition effects are greater than those experienced under interspecific competition (i.e., co‐existence theory; Adler et al. 2018; Chesson 2000). This suggests that sympatry between invasive species may be in part achievable when interspecific competition effects do not appreciably add to those already exerted by intraspecific competition.

Our findings carry broad implications for how invasive species interactions are studied and interpreted. That the same species pairs can shift between facilitation and competition depending on density and species ratios highlights the value of multi‐density experimental designs in capturing the full complexity of co‐invader relationships. Future work should manipulate resource availability across wider density gradients to identify the directionality of interaction outcome thresholds, use species of more comparable body size to decouple size‐asymmetry effects and control for kin structure—which gregarious larval production prevented here for *C. stygia* —given that kin recognition can promote larval cooperation in some Diptera (Khodaei and Long 2019). Longer‐term studies tracking species interactions and population dynamics in field settings, where density can fluctuate considerably, will be essential for translating our laboratory findings into real‐world predictions. As biological invasions escalate globally and bring increasing numbers of ecologically similar species into contact, our results suggest that neither competition nor facilitation should be assumed as the default outcome of co‐invader interactions.

## Author Contributions

**Daniel Nunn:** conceptualization (equal), data curation (lead), formal analysis (lead), investigation (lead), methodology (lead), visualization (lead), writing – original draft (lead), writing – review and editing (equal). **Nathan J. Butterworth:** conceptualization (supporting), formal analysis (supporting), supervision (supporting), writing – review and editing (equal). **Angela McGaughran:** conceptualization (equal), funding acquisition (lead), project administration (lead), resources (lead), supervision (lead), writing – review and editing (equal).

## Funding

This project was supported by the Royal Society Te Apārangi Marsden Fund (MFP‐UOW2302 to A.M.).

## Disclosure

Statement on Inclusion: Our study was a collaboration between researchers in New Zealand and Australia. The study species are prevalent in both countries, so they are of interest to both parties. All sample collection from conservation reserves or private spaces was approved by the proper authorities.

## Conflicts of Interest

The authors declare no conflicts of interest.

## Data Availability

All data required to reproduce the analyses and figures in this manuscript are available at: https://github.com/invasomics/blowflies/tree/main/coinvader_densities.
